# Stochastic processes in development and disease

**DOI:** 10.1098/rstb.2023.0043

**Published:** 2024-04-22

**Authors:** Dagan Jenkins, Jonathan R. Chubb, Gabriel Galea

**Affiliations:** ^1^ Genetics and Genomic Medicine Programme, Great Ormond Street Institute of Child Health, University College London, London WC1N 1EH, UK; ^2^ Developmental Biology and Cancer Programme, Great Ormond Street Institute of Child Health, University College London, London WC1N 1EH, UK; ^3^ UCL Laboratory of Molecular Cell Biology, University College London, Gower Street, London WC1E 6BT, UK

## 1. Introduction

Stochastic processes impact upon many areas of biology and investigating them requires diverse approaches. In April 2023, a Hooke Discussion Meeting (Royal Society, London, UK) brought together scientists with backgrounds in genetics, developmental biology and cancer together with experts from more traditionally quantitative disciplines to combine viewpoints into moulding a sense of where the field is and where it might go. Sessions were organized around cell state and single-cell transcriptomics, mathematical modelling, genetic and epigenetic disease, and cellular signalling and canalization.

Much of the emphasis on stochastic processes in biology has been on gene expression, perhaps because the techniques to measure gene expression are accessible and widely used, rather than effects on gene expression being especially dominant compared to other aspects of cell physiology. Early work reporting seemingly random gene expression goes back at least to the 1950s [1], although the ‘field’ of stochastic gene expression had to wait until the early 2000s for a substantial surge in popularity [2]. A workshop on stochastic processes in development [3] was held in 2010, marrying then-new single-cell technology together with some new developmental phenomena that might have been explainable by stochastic gene expression as an underlying cause. The available technology included imaging methods to study gene expression dynamics in single cells and initial attempts to bring these approaches into both *in vitro* differentiation models and tissues. However, the subsequent development and extensive use of genome-wide single-cell approaches—primarily to measure single-cell transcriptomes, but more recently other features, such as chromatin state, RNA turnover and the spatial organization of transcript content in complex cell populations—suggested it would be timely to organize another meeting to discuss recent progress and current challenges in this area.

One persistent challenge that needed to be absorbed by the discussion was the fuzziness of nomenclature to describe different sources of stochasticity. The heterogeneity of gene expression in genetically identical cells exposed to the same conditions is often referred to as *gene expression noise*. A popular operational sub-division of noise into ‘intrinsic’ and ‘extrinsic’ noise [4] is still commonly used. *Intrinsic noise* describes the variation in the expression between two identical reporter genes within single cells, which is ascribed to the low numbers of molecules (e.g. transcription factors) with control functions on gene expression. By contrast, *extrinsic noise* reflects the variation in reporter expression between cells in otherwise identical conditions, which would represent, for example, different cell-cycle states, mitochondrial content or signalling histories. While extrinsic noise is generally perceived to have an underlying cause, the sheer complexity of distinguishing mechanisms involved means that extrinsic noise tends to be bundled into intrinsic noise, at least in modelling approaches. While the early experiments comparing reporters in microbes found clear separation of sources of variation, in more complex developmental and disease models, with many hidden variables, these definitions cannot be cleanly applied. The need for consistent use of definitions in different systems was debated and use of operational definitions without sufficient care can be misleading: intrinsic noise is often bundled together with transcriptional bursting—this may be convenient in modelling, but it ignores the reality that transcription is regulated by signalling. Along similar lines, it seems important to be clear whether a disease phenotype becomes penetrant because of molecular noise or some (albeit unknown) chemical or mechanical stress.

To generalize these examples, considering noise within biological systems—whether this be ‘real’ noise or a convenient approach to modelling complex deterministic processes—raises questions about where we are to find the clear *causal objects*. There is a tendency to look at the gene because there is so much data available at that level and discoveries of mutants for many genes that cause striking developmental and disease phenotypes anchored the gene-as-instructive view. However, variability among genetically identical individuals, ranging from cellular diversity within a multicellular organism to incomplete penetrance for complex morphological traits, forces us to acknowledge that genetic instructions interact with mechanisms and information within the wider system, such that for the most part, there is no privileged scale at which biological functions are determined [5].

## 2. Variable biological properties can influence biological function positively or negatively

A key challenge in studying probabilistic biological systems remains the identification of intrinsic noise when the measured output is often an emergent property assessed at different length scales and/or later timepoints with multiple potential causal objects. Robert Johnston and co-workers [6] illustrate this through elegant cell fate specification models, including rhodopsin choice by photoreceptors in the *Drosophila* eye. The proportion of cells which adopt *rhodopsin 4* expression is largely consistent between individuals, but their position is variable. However, the stochastic distribution of *rhodopsin 4* expression is deterministically controlled by prior expression of a transcription factor, *spineless*. Stochastic initiation of *spineless* expression acts as a binary switch, deterministically instructing differentiation. Thus, the stochastic positioning of *rhodopsin 4*-expressing cells is deterministically controlled by the binary expression of *spineless*, itself stochastically distributed by the action of as yet unknown earlier events.

A similar trans-scale problem is discussed in the pathological context of congenital malformations. Some congenital malformations correspond to a binary event earlier in development. For example, incomplete closure of the neural tube causes a portion of the central nervous system to remain exposed on the back of the embryo, causing neural tube defects. Even between isogenic individuals, these malformations are often ‘partially penetrant’—affecting some individuals but not others—and can be phenotypically variable, for example in the length of exposed neural tissue. Dagan Jenkins [7] discusses partial penetrance and phenotypic variability in the context of congenital limb malformations, proposing a genetic threshold model to explain their variable emergence. Limb malformations are a particularly tractable system to identify variable outcomes by comparing the left and right sides, which develop largely independently and often show different levels of pathology. Manuscripts in this special issue also discuss pathological contributions of stochastic processes related to cancer. Andrew Teschendorff [8] describes ‘quasi-stochastic’ DNA methylation changes which cells accrue during division and accumulate with age. He proposes an epigenetic ‘clock’ wherein accumulation of gene dysregulation, particularly suppression of tissue-specific transcription factors, increases cell plasticity and the probability of becoming cancerous. A review by Cristina Pina [9] considers stochastic gene expression as a promoter of cancer progression. She discusses a type of leukaemia in which loss of an epigenetic regulator increases transcriptional variability in leukaemic compared with pre-leukaemic cells. This variability can then allow exploration of different phenotypes making the cancer more robust and/or pernicious, for example, by allowing the persistence of rare cells when subject to drug treatment.

This epigenetic repression of gene expression noise suggests evolution of noise-buffering mechanisms. Carla Mulas [10] provides another example of transcriptional noise buffering: post-transcriptional modification of proteins to achieve rapid and coherent transitions in cell state during differentiation of stem cells, providing greater synchronicity than would be expected from the variable levels of their transcripts. Various presentations during the meeting also discussed buffering through redundancy; at the molecular level through feedback loops or complementary branches of gene regulatory networks, at the organelle level by sequestration of ‘excess’ proteins in phase separated compartments [11] and at the cellular level as exemplified by the dispensability of practically any individual cell during vertebrate morphogenesis. Effects at the level of the cell are considered by Pilar Guerrero & Ruben Perez-Carasco [12], who explore mechanical causes and consequences of biological variability, namely the effect of friction on cellular deformations, for example during mitosis.

Active buffering—or adaptation to functionally consequential biological noise—suggests that the sources of variability are either uncontrollable (e.g. ion diffusion rates) or potentially beneficial. James Locke and co-workers [13] review examples of beneficial stochastic variability in germination of isogenic seedlings as ‘bet-hedging’ against catastrophic environmental changes. Veronica van Heyningen [14] discusses further benefits of variability, such as stochastic DNA recombination which generates immunological diversity and the inherent need for genetic diversity to permit evolutionary adaptation. Also discussed in depth was the potential for noise to enable cellular plasticity—although likely a bad thing in a tumour, the potential for some random fluctuations may enhance the impetus for reversal of differentiation for a cell that finds itself in the wrong niche [15]. These and other examples emphasize the importance of quantifying variability in biological parameters as potentially beneficial or pathogenic.

## 3. Technology

One of the motivations for the meeting was the explosion in single-cell technologies in the past 10 years. Single-cell RNAseq is perhaps, in isolation, not especially good at measuring stochastic gene expression—the data are too noisy, and highly top-sliced, capturing accurate estimates of transcript abundance for only the most strongly expressed genes, although simulations indicate scRNAseq can certainly reflect the outputs of noisy transcription [16]. Rory Maizels [17] writes a thoughtful piece on how these technologies have been further developed and exploited to superimpose temporal information onto the data. Although data on specific genes in single cells may suffer from technical noise, aggregated information on similar cells is providing the potential to predict how differences (random or otherwise) between cells at one time point can map onto phenotypic differences later on in development (or cancer, infection, etc.). The inferred cell trajectories are only predictions, it would of course be useful to test these predictions, by following the individual cells in real time, while imaging their gene expression. In addition, with single-cell proteomics appearing on the horizon, we should also be concerned that much of the variability we observe at the transcript level may not be particularly strong at the protein level, at least in an initial survey [18]. Maizels finishes by implying that it is perhaps not the technology that is limiting us, but our own conceptual frameworks for defining causality.

## 4. Future outlook

An important outcome of the Hooke meeting and this special issue resulting from it is the raising of new questions. Stochastic effects have been invoked to explain processes ranging from the activity of individual ion channels to the emergence of cancer, yet the field is rarely brought together to compare notes. We hope that conversations started during this meeting will lead to rich interdisciplinary collaborations providing new insights into the causes and consequences of stochastic processes in biology.

## Data Availability

This article does not contain any additional data.
